# In Vitro Efficacy of *Thymbra capitata* (L.) Cav. Essential Oil Against Olive Phytopathogenic Fungi

**DOI:** 10.3390/microorganisms13071503

**Published:** 2025-06-27

**Authors:** Gabriele Simone, Margherita Campo, Silvia Urciuoli, Lorenzo Moncini, Maider Giorgini, Francesca Ieri, Pamela Vignolini

**Affiliations:** 1 Centro Ricerche Strumenti Biotecnici nel Settore Agricolo-Forestale (CRISBA), ISIS “Leopoldo II di Lorena” di Grosseto, Cittadella dello Studente, 58100 Grosseto, Italy; gabriele.s@hotmail.it (G.S.); moncini@crisba.eu (L.M.); maidergiorgini@hotmail.it (M.G.); 2 PHYTOLAB Laboratory, Department of Statistics, Informatics, Applications “G. Parenti” (DiSIA), University of Florence, Via U. Schiff 6, Sesto Fiorentino, 50019 Florence, Italy; margherita.campo@unifi.it (M.C.); pamela.vignolini@unifi.it (P.V.); 3 Institute of Bioscience and BioResources (IBBR), National Research Council of Italy (CNR), Sesto Fiorentino, 50019 Florence, Italy; francesca.ieri@ibbr.cnr.it

**Keywords:** carvacrol, *Arthrinium marii*, GC–MS analysis, *Fomitiporia mediterranea*

## Abstract

In recent years, the excessive use of pesticides has raised environmental and health concerns, which has led to research into natural alternatives. Essential oils may represent a sustainable solution to this problem. In this study, essential oils from *Thymbra capitata* (L.) Cav., *Eucalyptus globulus* Labill, and *Mentha piperita* L. were analyzed by GC–MS and tested in vitro using the poisoned food technique against six olive pathogen fungi: *Alternaria* sp., *Arthrinium marii*, *Colletotrichum acutatum*, *Fomitiporia mediterranea*, *Fusarium solani*, and *Verticillium dahliae*. *T. capitata* essential oil (0.1 g/L) showed the highest antifungal activity when compared to *E. globulus* and *M. piperita* essential oils, which exhibited significantly lower efficacy against the tested olive phytopathogenic fungi. GC–MS analysis revealed that carvacrol is the main compound (76.1%) in *T. capitata* essential oil. A comparison of the inhibitory effect of *T. capitata* essential oil (0.1 g/L) and carvacrol (0.07 g/L) on selected fungal strains showed similar results, with carvacrol slightly more effective, although the differences were mostly statistically insignificant, except for *C. acutatum*. To the authors knowledge, this is the first study demonstrating the inhibitory effect of *Thymbra capitata* essential oil against *A. marii* and *F. mediterranea.* The results of this study represent a basis for the development of new biochemical biopesticides based on *T. capitata* essential oil as a useful tool for the contrast of some fungal olive tree diseases.

## 1. Introduction

### 1.1. Background

In recent years, increasing attention has been paid by International Organizations and the scientific community to the use of pesticides in the agricultural and agro-industrial sectors due to environmental pollution, toxicological issues, and the development of resistance or cross-resistance caused by the excessive use of synthetic chemicals without adequate regulation. According to the FAO, pesticide use increased 70% between 2000 and 2022, with the Americas accounting for half of the global pesticide use in 2022 [1]. Taking up the challenges of the UN 2030 Agenda for Sustainable Development, the legislation on chemicals and pesticides started establishing harmonized restrictions on the marketing and use of hazardous substances and promoted their replacement with new products mostly based on natural active principles. Furthermore, greater consumer awareness has led to an increase in the organic production and market, with 96.4 million hectares of land dedicated to organic farming in 2022 vs. 15 million hectares in 2000, whereas the organic market increased from EUR 15.1 billion in 2000 to EUR 134.8 billion in 2022 [2]. On the other hand, despite the use of large amounts of biopesticides and conventional pesticides, the economy of the agricultural sector continues to be affected by the possible spread of pathogenic microbial species that limit the productivity of crops. This, also given the increased demand for products by the ever-growing population, determined a strong push for companies towards research to maximize the potential of biopesticides so that they can fully replace synthetic products without significantly affecting productivity. Essential oils (EOs) are complex mixtures that can contain over 200 individual volatile organic compounds (VOCs) in some instances. Each of these components plays a role in either the positive or negative effects of EOs. Consequently, having a deep understanding of the composition of EOs enables more effective and targeted applications. EOs are an interesting example of biochemical biopesticides, natural compounds able to control pests without significant toxicity mechanisms for humans and the environment, enabling the Sustainable Development Goals promoted by the UN 2030 Agenda to be met in terms of agro-yields, environmental safety, socio-cultural acceptability, and green technology employment [3]. Their major components belong to the chemical classes of terpenes, terpenoids, and aromatic or aliphatic compounds with low molecular weight and can constitute up to 85% of the EO composition. Limonene, α-phellandrene, carvacrol (CAR), thymol, α/β-thujone, carvone, menthol, and menthone are among the most represented compounds [4]. The antimicrobial activity of EOs was largely demonstrated against different fungi and bacterial pathogens and also towards some drug-resistant or multidrug-resistant strains, revealing interesting antimicrobial action for most of the tested species depending on the EO dose, even though most of the studies concerned pathogens of biomedical interest [5,6,7,8]; the mechanism of action is mainly based on membrane damage or alterations to its permeability, cell wall damage, or modifications of the cytoplasm for bacteria, while, in *Aspergillus flavus* and *Candida* isolates, inhibition of ergosterol biosynthesis was observed [4]. Previous studies demonstrated the efficacy of EOs obtained from several *Eucalyptus* species (including *Eucalyptus globulus* Labill (*E. globulus*)) and *Mentha piperita* L. (*M. piperita*), both in vitro and in vivo, towards microbial strains of agricultural interest, such as *Alternaria solani*, *Alternaria alternata*, *Alternaria arborescens*, *Botrytis cinerea*, *Colletotrichum gloeosporioides*, *Colletotrichum acutatum*, *Penicillium expansum*, and *Verticillium dahliae*. As an example, *M. piperita* EO (MpEO) showed antifungal activity in vitro against *Verticillium dahliae,* with a minimum inhibitory concentration of 100 μL/mL and minimum fungicidal concentration of 150 μL/mL, whereas *E. globulus* EO (EgEO), tested against *Alternaria solani*, showed inhibition of mycelial growth and spore germination, and it was able to control disease severity in tomato plants at concentrations between 2.0 and 10.0 μL/mL. The mechanisms of action, when specified, appear to be those typical of the EOs reported above [9,10,11,12,13,14,15,16,17,18]. *Thymbra capitata* (L.) Cav. EO (TcEO), for which fewer studies are available, showed antimicrobial activity in *Botrytis fabae* management thanks to its main constituents, such as CAR, 4-Methoxystyrene, and cyclohexene [19]; in the biomedical sector, its effectiveness was proven on methicillin-resistant *Staphylococcus aureus* biofilms, *Candida* and *Aspergillus* clinical strains, and dermatophytes like *Microsporum canis*, *Microsporum gypseum*, *Trichophyton rubrum*, *Trichophyton mentagrophytes,* and *Epidermophyton floccosum* [20,21].

### 1.2. Aim of the Study

The present study focused on the chemical characterization and evaluation of the in vitro inhibitory effect of EOs from *Thymbra capitata* L. (*T. capitata*), *E. globulus*, and *M. piperita* against *Alternaria* sp., *Arthrinium marii* (*A. marii*), *Fomitiporia mediterranea* (*F. mediterranea*), *Fusarium solani* (*F. solani*), and *Verticillium dahliae* (*V. dahliae*), phytopathogenic fungi of agronomical interest, with different infective processes and symptoms caused, particularly affecting olive tree (*Olea europaea* L.), one of the most widespread, economically important, and representative crops in the Mediterranean basin countries, with Italy placed second in terms of cultivated area in the European Union [22]. Moreover, the three EOs were characterized by their VOC content through quali-quantitative analysis by Gas Chromatography coupled with Mass Spectrometry detection (GC–MS) to obtain their complete profiles of volatile secondary metabolites. Then, the samples were tested for their inhibitory effects at the same concentration, chosen according to the commonly employed doses of EOs in organic plant protection products, to compare their antimicrobial activities against the selected fungal strains. TcEO, which showed the highest efficacy, was selected to test its inhibitory effect at different concentrations to assess the possible dose–effect relationships on the pathogens. In the last experiment, TcEO was tested in comparison with the pure standard of its main compound, CAR. In fact, the available evidence in the literature concerns the antimicrobial activity of CAR both as a single compound and as the main VOC in the composition of EOs obtained from several vegetal species, but the mechanism of action was investigated for only CAR, hypothesizing cell wall breakdown mainly due to permeability alterations for bacteria or the inhibition of ergosterol biosynthesis, alterations in protein folding ability, and endoplasmic reticulum stress for fungi [23,24,25,26]. Given this evidence and chemical analysis, our test aimed to assess the hypothesis that the effect of TcEO was mainly due to CAR, with a future perspective, eventually, of investigating possible synergies between CAR and the other components of the EO. The results of this study represent a contribution to the research that valorizes a spontaneous plant, which grows on marginal areas of the Mediterranean basin, as an alternative source for natural active principles that could be used as ingredients for new sustainable, non-toxic, and eco-friendly products that can effectively replace synthetic chemicals in green agriculture.

## 2. Materials and Methods

### 2.1. Chemicals

All analytical standards and reagents were purchased from Sigma Aldrich (Steinheim, Germany). A mixture of linear alkanes (C10–C26), heptane, and tridecane in hexane was used. Helium and nitrogen (99.999%) were purchased from SOL Gas (Monza, Italy).

### 2.2. Essential Oils

The EOs used in this study are extracted from *E. globulus*, *M.piperita*, and *T. capitata* plants. All EOs were provided free of charge by the company E.Lab (Pistoia, Italy). The plant materials of *E. globulus* and *M. piperita* were sourced from Tuscany (Italy), while *T. capitata* originated from Sicily (Italy). All plants were cultivated following organic farming practices.

### 2.3. GC–MS Analysis

The EO samples were diluted (1:10,000) with heptane, in which tridecane (20 ppm) was present as internal standard. GC–MS analysis of EOs was performed using an Agilent 7820a Gas Chromatograph equipped with a 5977e MSD quadrupole mass spectrometer operating in split-less mode (Agilent Technologies, Palo Alto, CA, USA). Further, 1 μL of each sample was injected. A Gerstel MPS2 XL autosampler (Gerstel, Mülheim an der Ruhr, Germany) equipped with liquid option was used. The analyte separation was performed with an Agilent DB InnoWAX column (50 m, 0.20 μm i.d., and 0.40 μm o.d.). Injector temperature was 260 °C, and helium was used as carrier gas (flow rate: 1.2 mL/min; pressure: 33 psi). The separation was carried out according to the following method: initial oven temperature 40 °C, for 1 min, then increased to 200 °C (5 °C/min), finally to 250 °C (10 °C/min), and held at 240 °C for 6 min. The Total Ion Current chromatograms were recorded: the mass spectrometer range was 29–330 *m*/*z*, with an electron ionization of 70 eV, at three scans/s. The compounds were tentatively identified by comparing the mass spectra of each peak with those reported in the official mass spectral database NIST MS library (NIST14); when available, peak identification was confirmed by authentic standard analysis under the same conditions. The identification of the peaks was confirmed by comparing their retention index: a mixture of linear alkanes (C10–C26) in hexane was then injected under the conditions described for the sample analysis, and the retention indices were calculated by the generalized equation [27] and compared with the literature [28] The relative concentration of each identified compound was calculated as peak area on total area of all the identified peaks (peak areas were normalized using tridecane as internal standard). Data are the means of three determinations (SD < 5%).

### 2.4. Fungal Strains

The test was carried out using the following fungal isolates: *Alternaria* sp. UNIPI 1189 (Mycologic Laboratory of Plant Pathology of the Agricultural Science, Alimentary and Agro-environmental Department, University of Pisa, Pisa, Italy), *A. marii* DiSSPA_AP1 (Department of Soil, Plant and Food Sciences (DiSSPA), University of Bari Aldo Moro, Bari, Italy), *C. acutatum* F64 (Phytoparasites Diagnostics (PhyDia) s.r.l., Via San Camillo de Lellis, snc, Viterbo, Italy), *F. solani* A209, *F. mediterranea* A125, and *V. dahliae* A127 (Plant Pathology Section, Department of Agricultural, Food, Environmental and Forestry Science and Technology, University of Florence, Firenze, Italy), all known to be pathogens of *Olea europaea* L. The strains were grown on 90mm Petri dishes on potato dextrose agar (PDA), preserved in fridge at 4 °C for long-term storage, or in incubator at 25 °C for active growth.

### 2.5. Substrates

All the substrates are based on potato dextrose agar (PDA) eventually enriched with the tested substance. The PDA was prepared as a label, sterilized by autoclaving it at 121 °C for 21 min, and poured in 90 mm Petri dishes. The different oils were added after the sterilization directly into the bottle, as soon as the molten substrate cooled to around 50 °C, using a micropipette with sterilized tips and shaking it vigorously afterward.

### 2.6. Test Design

#### 2.6.1. First Screening

In the first test, the objective was to conduct a general screening of the EOs’ effects against the selected pathogens using a single dose of multiple EOs (EgEO, MpEO, and TcOE.). The dose selected is 0.1 g/L, compared with the control composed of plain PDA without any other additions. The EO with the highest inhibition rate was then selected for the following test.

#### 2.6.2. Dose Test

In the second test, TcEO (the best-performing in the previous experiment) was tested at different concentrations to determine the optimal dose for the total inhibition of the pathogens. The selected doses were 0 g/L (control), 0.05 g/L, 0.1 g/L, 0.2 g/L, and 0.3 g/L. After the end of the test, the plates without any myceliar growth were used for a second sub-test to determine if the effect of the EO was fungistatic or fungitoxic. The myceliar plugs were transferred to a PDA plate and incubated at 25 °C for a week. If no further growth occurred, the dose was considered fungitoxic; on the contrary, growth resuming was considered caused by a fungistatic effect.

#### 2.6.3. Carvacrol Comparison

The test was completed by comparing the action of TcEO with its most representative component, CAR, to verify if the inhibitory effect on olive tree phytopathogens was exclusively due to this. Therefore, the growth of each phytopathogen on the control substrate (CTRL) was compared with the basic dose of TcEO 0.1 g/L and its corresponding dose in CAR, 0.07 g/L.

### 2.7. Test Execution

All three tests were carried out using the poisoned food technique [29]: the substrate is enriched with the tested substance and is inoculated with a chosen pathogen; the growth is compared with the colony grown in a substrate without additions. The inhibition value is calculated by comparing the colony diameter at the end of the test.

The inoculum was obtained from the border of a colony in active growth, using a 6 mm cork borer to obtain myceliar plugs that are then placed in the center of each Petri dish. The inoculated dishes were incubated in darkness at 25 °C. When at least one of the colonies arrives at the border of the dish, the test is stopped, and the diameters are measured. The inhibition value was calculated with the formula

Percentage inhibition = [Dc * Dt)/Dc] * 100

Dc = diameter of the control thesis, and Dt = diameter of the treated thesis [30]:

The half-maximal effective concentration EC50 was calculated using GraphPad Prism^®^ version 10.4.2. Each test is repeated two times, and each thesis was conducted in triplicate.

### 2.8. Statistical Analysis

The statistical analysis was carried out using the Excel macro DSAASTAT using the analysis of variance (ANOVA). The mean was separated using the Fisher Least Significant Difference (LSD) multiple comparison test, preceded by the angular transformation of the percentual values. The statistical significance was evaluated with *p* < 0.05 [31].

## 3. Results

### 3.1. GC–MS Analysis of EOs

EgEO, MpEO, and TcEO were analyzed by GC–MS, and the chemical compositions are summarized in Table 1. A total of 33 different VOCs were identified in the three OEs. Eucalyptol, also called cineole, was by far the main component (87%) of EgEO, menthol (41% as a sum of isomers) and menthone (25%) were present in MpEO, and CAR was the most abundant in TcEO, with a high content of 75%. CAR is a phenolic monoterpenoid found in OEs of oregano (*Origanum vulgare*), thyme (*Thymus vulgaris*), and other plants. Even TcOE is reported to be mostly of the CAR type, and this has also been confirmed by our GC–MS analyses [20]. Among the compounds present in lower amounts in the EgEO were monoterpene hydrocarbons α-pinene and limonene, with percentages ranging from 2% to 4%. Oxygenated monoterpene α-terpineol also reached a percentage of about 3%. Oxygenated monoterpene cineole was one of the main minor compounds found in MpEO (7%), along with menthyl acetate, limonene, and the sesquiterpene β-caryophillene, each present in relative abundances ranging from 3% to 4%. Aromatic monoterpene p-cymene was the main constituent of TcEO after CAR, of which it is the precursor (13%). β-caryophillene followed with a percentage of 3%, and oxygenated monoterpene linalool with about 2%. The isomer of CAR, thymol, was found in low amounts in this EO (1%), confirming the CAR-type nature of TcEO.

### 3.2. First Screening and Selection of TcOE

For the initial screening, the TcEO dose of 0.1 g/L was selected based on previous tests conducted with TcEO on phytopathogens, as outlined in a patent by Morana et al. [32]. The same dose was applied to EgEO and MpEO for comparison with TcEO (Table 2).

The three selected EOs were evaluated against a panel of phytopathogens. EgEO and MpEO showed the least effectiveness, with negligible inhibitory effects. The maximum effect observed with eucalyptus EO was against *A. marii*, with an inhibition of 13.20% but with a high standard deviation, while with mint oil the maximum effect was against *Alternaria* sp., with 10.30% inhibition. On the contrary, interesting results were observed with the TcOE. The lowest inhibition effects were against, respectively, *F. mediterranea* (28.93%), *C. acutatum* (35.85%), and *F. solani* (47.04%). The TcOE was more effective against *Alternaria* sp. (66.05%) and *V. dahliae* (68.89%), while against *A. marii* the inhibition was nearly total (99.57%). Of these three tested substances, TcEO was the most effective and was selected for the following trials.

### 3.3. TcEO Dosage Test

**Figure 1:**
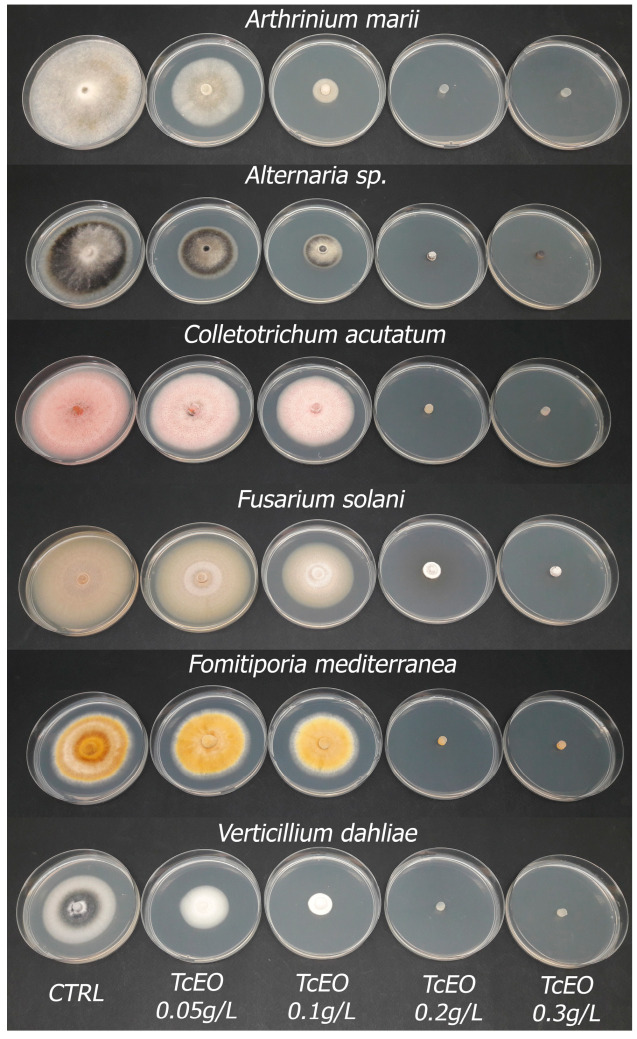
Inhibition of *Thymbra capitata* essential oil (TcEO) tested at different doses against selected fungal strains. The photos were created using the most representative Petri dish among 6 replicates.

The results of the first screening led to the selection of TcEO, which was then tested at various doses to assess its inhibition percentage against the chosen phytopathogens (Table 3). In all the cases, we can observe an increasing antifungal effect proportional to the substance dose, up to 100% at the maximum dose in most of the cases (Figure 1). A lower inhibition effect was observed against *F. solani*, with an effectiveness of 8.27% at a half dose, 25.24% at a full dose, 75.12% at a double dose, and 93.50% at a triple dose. The rise in effectiveness was more sharp in *C. acutatum*, where, in the first two doses, the inhibition was low (18.78% and 25.71%) but then rose to 97.85% and 100% with the double and triple dose. The same trend, but even more pronounced, was observed with *F. mediterranea*. The inhibition of the first two doses was very low (6.46% and 12.06%) but rose abruptly to 100% at the double dose. The effect against *Alternaria* and *V. dahliae* was very similar, following a common pattern. The half dose provided an inhibition of 24.39% against *Alternaria* and 29.16% against *V. dahliae*. Doubling the dose, the inhibition percentage rose by almost double (53.69% in *Alternaria* and 65.40% in *V. dahliae*). A similar trend was observed when the dose was doubled again, reaching just below 100% inhibition. The best results were achieved against *A. marii*, where even the half dose inhibited myceliar growth by 58.49%, reaching 89.57% at 0.1 g/L and 100% inhibition at higher doses. The EC_50_ values ranged from 0.05 g/L with *A. marii* to 0.14 g/L for *F. solani*. When the inhibition reached 100%, the myceliar plugs were replated on new fresh PDA to test an eventual fungitoxic effect. *Alternaria* sp. and *C. acutatum* were only inhibited by the TcEO, but, on the contrary, a fungitoxic effect was observed against *A. marii*, *F. mediterranea*, and *V. dahliae*.

### 3.4. Comparison Between TcEO and CAR

**Figure 2:**
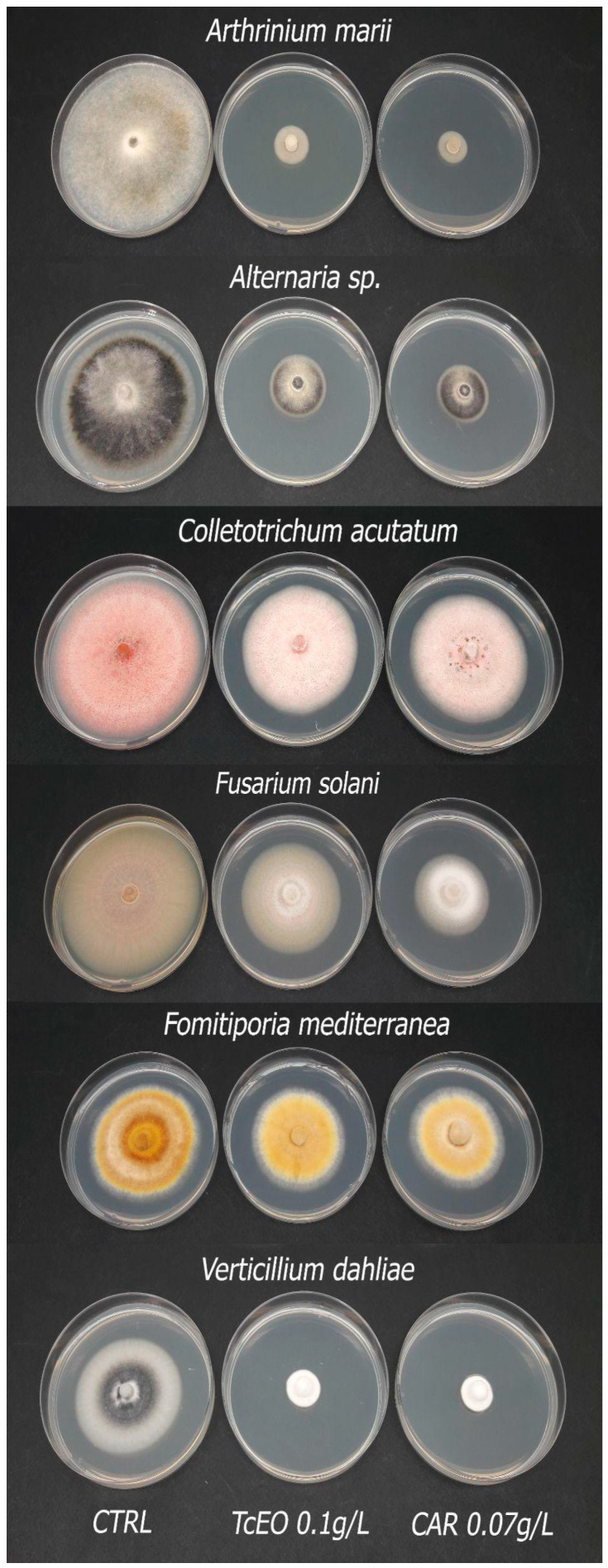
Comparison of the inhibition of *Thymbra capitata* essential oil (TcEO) and carvacrol (CAR) against the selected fungal strains. The photos were created using the most representative Petri dish among 6 replicates.

The GC–MS analyses of TcEO showed that the main compound is carvacrol. Based on this evidence, we decided to compare the inhibitory effect on the selected fungal strains of TcEO at a concentration of 0.1 g/L and CAR at a concentration of 0.07 g/L, which corresponds to the amount present in the analyzed TcEO (Table 4). In all the tested fungi, we observed that the inhibition was the same between the two samples. Carvacrol was a little bit more effective than the entire essential oil, but almost all the differences were statistically insignificant, except with *C. acutatum*, where the means can be separated, but the percentages remained very close (Figure 2).

## 4. Discussion

Essential oils (EOs) are complex mixtures of VOCs, including terpenes, terpenoids, and aromatic or aliphatic compounds with low molecular weight. These compounds can have synergistic and modular actions that are responsible for the overall antimicrobial activity of the oil. The chemical composition, highly variable depending on the plant origin, therefore represents a crucial element for the selection and targeted use of an EO in phytosanitary control. GC–MS analysis allowed us to obtain the complete profile of volatile secondary metabolites of three EOs, enabling authentication, quality, and relative abundance of major and minor compounds exhibiting potential antimicrobial activity. In particular, cineole was by far the main component of EgEO (87%) but was also present in MpEO (7%) and in a low quantity in TcEO. Menthol, menthone, and menthyl acetate were found only in MpEO, instead of compounds such as α-pinene, limonene, p-cymene, and 4-ol-terpinen, which were present in the three EOs in percentages ranging from 0.5% to 13%. β-caryophillene was in MpEO and TcEO at about 3% but not in EgEO. Seventeen terpenes were present at less than 1% in all three EOs. Phenolic monoterpenoid CAR was only in TcEO, where it reached over 76% of the total volatile compounds.

A very low dose of 0.1 g/L was initially tested for all the EOs. This concentration proved effective for TcEO but not for EgEO and MpEO. In the literature, several studies have investigated the effectiveness of eucalyptus EO against phytopathogens. For instance, Hajji-Hedfi et al. reported up to 78.37% inhibition against *Colletotrichum gleosporioides* and 72.86% inhibition against *Alternaria alternata* with a dose of 4 µL/mL, approximately 40 times the dose used in this study [18]. Gakuubi et al. tested *Eucalyptus camaldulensis* EO against five *Fusarium* species, achieving complete inhibition of the myceliar growth with a dose of 7–8 µL/mL [33]. A third example is provided by Kottearachchi et al., where EOs of three *Eucalyptus* species were tested against *Sclerotium rolfsii* and *F. solani*, with a minimum inhibition dose ranging from 0.1% to 0.75% according to the pathogen and extract [34]. In all the cases, the doses were much higher than the one used in this work, suggesting that the findings here are consistent with previous research. A similar conclusion can be drawn for mint EO based on the following examples. Akhlaghi et al. evaluated a minimum inhibitory concentration (MIC) and a minimum fungicidal concentration (MFC) of *M. piperita* against *V. dahliae* at doses of 100 and 150 µL/mL [16]. Morkeliūnė et al. observed 88% inhibition against *C. acutatum* with a dose of 1600 µL/L of the same extract [15]. Finally, França et al. tested *M. piperita* extract against *Alternaria alternata* at doses ranging from 0 to 1%, with a maximum inhibition value of 41.5% [14]. Respectively, in these three works cited as examples, they observed inhibition at doses of approximately 100 g/L, 1.6 g/L, and 10 g/L, which are very high compared to the 0.1 g/L dose used in this work. The results with TcEO are particularly interesting as it inhibited mycelium growth at very low doses. An increase in inhibition, proportional to the dose increase, was observed for all the pathogens. At the lowest dose (0.05 g/L), the effects were minimal, with the mycelial growth almost comparable to the control, and there was a high standard deviation. It is hypothesized that, due to the volatility of TcEO and the small quantities used, even minor differences in substrate preparation between repetitions could lead to different results. At higher doses, TcEO is more effective, achieving 100% inhibition at a dose of 0.2 g/L against *A. marii* and *F. mediterranea*, and at 0.3 g/L against the other pathogens except for *F. solani*. These results are consistent with the literature findings. For example, Morkeliūnė et al. observed the complete inhibition of *C. acutatum* with a dose of 200 µL/L of TcEO, which is close to the 0.2 g/L dose used in this study [15]. Duduk et al. observed the total inhibition of *C. acutatum* at a concentration of 667 µL/L of TcEO, while Varo et al. reported the complete inhibition of two *V. dahliae* strains with a 5 g/L dose of the same extract [35,36]. The results for *Alternaria* sp. are also confirmed by studies such as Aslam et al., where doses of 0.2, 0.4, and 0.6 g/L of thyme EO (*Thymus vulgaris*, with a low carvacrol content) inhibited growth by 44.44%, 57.03%, and 70.81%; or by Saoud et al., who used concentrations from 0.1 to 0.5 µL/mL of TcEO with up to 100% inhibition at higher doses [37,38]. To our knowledge, no studies have investigated the inhibitory effect of TcEO against *A. marii* and *F. mediterranea*. For the third test, the aim was to compare the effectiveness of TcEO with CAR alone. The dose of carvacrol used (0.07 g/L) corresponded to the amount present in a 0.1 g/L dose of TcEO. Among the three possible outcomes (TcEO > CAR, TcEO = CAR, and TcEO < CAR), we consistently observed a comparable level of inhibition. This suggests that carvacrol is the main active compound in TcEO, with the slight differences between treatments proving statistically insignificant. The mechanism of action is still not fully elucidated, but there is a consensus in the literature that carvacrol can cause alteration and damage to the cell membrane, with consequent leakage of its content [39]. These alterations include an increase in membrane permeability and the disruption of the functions of the enzyme-mediated processes [40], a reduction in the membrane pH gradient with subsequent ATP depletion, and interactions with enzymes related to cell wall synthesis and molecule degradation [41]. The obtained results suggest the necessity of further in-depth studies to investigate the possibility of using TcEO to obtain new sustainable biopesticides or products for plant disease prevention. The volatility of the active ingredients contained in the EOs represents a critical factor in the formulation of products for agriculture, influencing not only their composition but also the persistence of the products after administration. Recent studies have demonstrated the usefulness of nanoparticles functionalized with EOs to stabilize the volatile active ingredients, preserving them from degradation and evaporation and increasing their compatibility with the other components of the formulation. The use of encapsulated formulations would also allow control of their release, enhancing their bioavailability and efficacy [4]. As regards the extraction of EOs on a large scale, in the literature, there are no recent studies reported on TcEO, but many works are available regarding other aromatic and medicinal species, such as thyme, peppermint, oregano, rosemary, lavender, or waste such as fruit peels. In addition to traditional methods such as steam extraction, there are non-conventional methodologies that have proven to be less expensive, time- and energy-saving, and that require the use of smaller quantities of solvent; all these can be tested for optimizing the extraction of TcEO [42].

## 5. Conclusions

The GC–MS analyses and in vitro tests performed in this study allowed us to describe and compare three EOs concerning their chemical composition and antimicrobial activity on one panel of olive fungal pathogens. TcEO proved to be active on the tested microorganisms at much lower doses than eucalyptus and mint EOs, producing inhibitions of up to 100% at a concentration of 0.2 g/L, which enabled selecting it for the following experiments. Hence, CAR was identified as both its main constituent and the main agent responsible for the antimicrobial action. The antimicrobial activity of the phytocomplex has proven comparable to that of CAR, allowing us to hypothesize the use of TcEO instead of purified carvacrol for the formulation of new biopesticides; this determines an advantage from an economic and environmental point of view since the large-scale production of carvacrol as a single compound requires specific purification processes. The sustainability of TcEO is also due to the fact that low amounts are sufficient to obtain high inhibition, a positive factor also given the low quantities of EO that are generally obtained by extraction from leaves compared to the demand of large-scale production. According to the authors’ knowledge, TcEO has been tested for the first time for its inhibitory activity towards *A. marii* and *F. mediterranea*, showing higher inhibitions than EgEO and MpEO, with EC_50_ values of 0.05 ± 0.03 g/L and 0.13 ± 0.05 g/L for *A. marii* and *F. mediterranea*, *respectively*. In light of these considerations, the results of this study represent a basis for the development of new biochemical biopesticides based on TcEO as a useful tool for the contrast of some fungal diseases of olive trees, whose use will have to be further investigated in field studies to confirm its potential in the eco-sustainable defense of the crop.

## Figures and Tables

**Table 1:** Volatile organic compounds (VOCs) in *E. globulus* essential oil (EgEO), *M. piperita* essential oil (MpEO), and *Thymbra capitata* essential oil (TcEO) identified by GC–MS analysis. For each compound, concentration is expressed as area % of the total area after normalization with internal standard. Data are the mean of three determinations (SD < 5%). Retention indices calculated (RIcal): non-isothermal Kovats retention indices [27]. Retention indices reference (RIref): non-isothermal Kovats retention indices from ChemistryWebBook [28].

Compound	RIcal	RIref	EgEO	MpEO	TcEO
α-pinene	1030	1026	2.3	1.0	0.8
α-thujene	1030	1030	0.0	0.0	0.3
camphene	1067	1065	0.0	0.0	0.1
β-pinene	1122	1118	0.1	1.2	0.1
sabinene	1130	1126	0.0	0.7	0.0
myrcene	1168	1167	0.2	0.4	0.6
α-phellandrene	1177	1177	0.3	0.2	0.0
α-terpinene	1180	1179	0.3	0.5	0.4
2,3-dehydro-1,8-cineole	1202	1197	0.1	0.0	0.0
limonene	1185	1199	3.8	3.0	0.4
1,8-cineole	1225	1221	86.7	7.0	0.1
β-ocimene	1235	1245	0.3	0.5	0.0
γ-terpinene	1268	1254	0.3	0.5	0.2
p-cymene	1286	1281	1.2	0.6	13.3
terpinolene	1290	1305	0.0	0.3	0.0
allo-ocimene	1383	1377	0.1	0.0	0.0
germacrene D	1410	1409	0.0	2.0	0.0
menthone	1470	1476	0.0	25.5	0.0
isomenthone	1480	1484	0.0	4.2	0.0
linalool	1545	1544	0.1	0.4	1.6
menthyl acetate	1555	1561	0.0	4.0	0.0
4-ol-terpinen	1617	1612	0.5	1.7	1.4
β-caryophillene	1631	1625	0.0	3.6	3.0
menthol isomer	1610	1644	0.0	5.3	0.0
menthol isomer	1641	1644	0.0	35.9	0.0
alloaromandrene	1640	1645	0.3	0.0	0.0
α-humulene	1658	1667	0.0	0.5	0.1
α-terpineol	1675	1695	2.7	0.0	0.2
citral	1698	1695	0.1	0.0	0.0
(-)borneol	1706	1704	0.0	0.0	0.5
piperitone	1705	1710	0.0	0.9	0.0
α-terpinyl acetate	1722	1721	0.7	0.0	0.0
thymol	2155	2154	0.0	0.1	0.9
carvacrol	2230	2225	0.0	0.0	76.1

**Table 2:** First screening of the percentage (%) inhibition effects of essential oils (EOs) against selected fungal strains. *E. globulus* Labill. essential oil (EgEO); *M. piperita* L. essential oil (MpEO); *T. capitata* L. essential oil (TcEO). For each line, different letters are related to statistically different percentages according to Fisher LSD multiple comparison test after angular transformation of the mean values; *p* < 0.05.

Fungal Strains	Percentage (%) of Inhibition		
	**EgEO 0.1 g/L**	**MpEO 0.1 g/L**	**TcEO 0.1 g/L**
*Alternaria* sp.	3.60 ± 4.59 a	10.3 ± 3.25 b	66.05 ± 2.47 c
*Arthrinium marii*	13.20 ± 11.83 b	1.30 ± 1.64 a	99.57 ± 1.06 c
*Colletotrichum acutatum*	4.94 ± 1.98 a	7.71 ± 2.28 b	35.85 ± 5.54 c
*Fomitiporia mediterranea*	−0.41 ± 6.34 a	5.10 ± 4.68 b	28.93 ± 3.60 c
*Fusarium solani*	2.25 ± 3.27 a	2.82 ± 3.15 a	47.04 ± 15.04 b
*Verticillium dahliae*	−1.63 ± 2.93 a	1.60 ± 2.93 b	68.89 ± 4.78 c

**Table 3:** Percentage (%) of inhibition of *Thymbra capitata* essential oil (TcEO) tested at different doses against selected fungal strains and half-maximal effective concentration (EC50) values. For each line, different letters are related to statistically different percentages according to Fisher LSD multiple comparison test after angular transformation of the mean values; *p* < 0.05.

Fungal Strains	Percentage (%) of Inhibition				
	TcEO 0.05 g/L	TcEO 0.10 g/L	TcEO 0.20 g/L	TcEO 0.30 g/L	EC_50_ g/L
*Alternaria* sp.	24.39 ± 21.97 a	53.69 ± 12.19 b	98.27 ± 1.06 c	100.00 ± 0.00 c *	0.09 ± 0.06
*Arthrinium marii*	58.49 ± 23.27 a	89.57 ± 8.48 b	100.00 ± 0.00 c **	100.00 ± 0.00 c **	0.05 ± 0.03
*Colletotrichum acutatum*	18.78 ± 1.52 a	25.71 ± 0.61 b	97.85 ± 3.93 c	100.00 ± 0.00 d *	0.128 ± 0.005
*Fomitiporia mediterranea*	6.46 ± 2.19 a	12.06 ± 3.30 b	100.00 ± 0.00 c **	100.00 ± 0.00 c **	0.13 ± 0.05
*Fusarium solani*	8.27 ± 6.14 a	25.24 ± 14.17 b	75.12 ± 14.25 c	93.50 ± 5.80 d	0.14 ± 0.01
*Verticillium dahliae*	29.16 ± 8.99 a	65.40 ± 5.88 b	97.06 ± 3.60 c	100.00 ± 0.00 d **	0.08 ± 0.03

* Fungistatic effect; ** fungitoxic effect.

**Table 4:** Comparison of the inhibition percentages (%) of *Thymbra capitata* essential oil (TcEO) and carvacrol (CAR) against the selected fungal strains. For each line, different letters are related to statistically different percentages according to Fisher LSD multiple comparison test after angular transformation of the mean values; *p* < 0.05.

Fungal Strains	Percentage (%) of Inhibition	
	TcEO 0.10 g/L	CAR 0.07 g/L
*Alternaria* sp.	53.69 ± 12.19 a	60.16 ± 11.17 a
*Arthrinium marii*	89.57 ± 8.48 a	91.67 ± 9.86 a
*Colletotrichum acutatum*	25.71 ± 0.61 a	28.24 ± 1.25 b
*Fomitiporia mediterranea*	12.06 ± 3.30 a	6.95 ± 3.00 a
*Fusarium solani*	25.24 ± 14.17 a	26.86 ± 15.54 a
*Verticillium dahliae*	65.40 ± 5.88 a	65.79 ± 10.51 a

## Data Availability

The original contributions presented in this study are included in the article. Further inquiries can be directed to the corresponding author.

## Abbreviations

The following abbreviations are used in this manuscript:

CAR	Carvacrol
CTRL	Control
EOs	Essential Oils
GC	Gas Chromatography
EgEO	*Eucalyptus globulus* Labill. Essential Oil
LSD	Least Significant Difference
MFC	Minimum Fungicidal Concentration
MIC	Minimum Inhibitory Concentration
MpEO	*Mentha piperita* L. Essential Oil
MS	Mass Spectrometry
PDA	Potato Dextrose Agar
RIcal	Retention Indices Calculated
RIref	Retention Indices Reference
TcEO	*Thymbra capitata* Essential Oil
VOCs	Organic Volatile Compounds
